# Digitoxin activates EGR1 and synergizes with paclitaxel on human breast cancer cells

**DOI:** 10.4103/1477-3163.72578

**Published:** 2010-11-18

**Authors:** Linda Saxe Einbond, Hsan-au Wu, Tao Su, Tangel Chang, Maya Panjikaran, Xiaomei Wang, Sarah Goldsberry

**Affiliations:** Columbia University College of Physicians and Surgeons, New York, NY, USA; 1 Department of Oncological Sciences, Mount Sinai School of Medicine, New York, NY, USA; 2 Western University of Health Sciences, Pomona, CA, USA; 3 Regeneron Pharmaceuticals, Tarrytown, NY, USA; 4 Keck School of Medicine, University of Southern California, Los Angeles, CA, USA

**Keywords:** Cardiac glycosides, microarrays, paclitaxel, stress response, synergy

## Abstract

**Background:**

Numerous studies have suggested that digitalis derivatives promise to be superior to existing adjuvant therapy for breast cancer as to effects and side-effects. In the present study, we have used gene expression analysis to determine the molecular action of digitoxin on breast cancer cells and assessed digitoxin’s ability to synergize with the chemotherapy agent paclitaxel with respect to inhibition of cell proliferation

**Materials and Methods:**

We treated (Her2 overexpressing, ER low) MDA-MB-453 human breast cancer cells with digitoxin at four doses {20 ng/ml (26 nM) to 1 μg/ml} and collected RNA at 6 h and 24 h for gene expression analysis. To examine the effects on ER positive cells, we treated MCF7 cells with digitoxin at 1 μg/ml and collected RNA for RT-PCR analysis. In addition, we assayed the growth inhibitory effect of low doses of digitoxin combined with paclitaxel and determined combination index values.

**Results:**

To reveal primary effects, we examined digitoxin’s effect 6 h post-treatment with the highest dose, 1μg/ml, and found upregulation of the stress response genes EGR-1 and NAB2, lipid biosynthetic genes and the tumor suppressor gene p21, and downregulation of the mitotic cell cycle gene CDC16 and the replication gene PolR3B. RT-PCR analysis validated effects on stress response, apoptotic and cell cycle genes on MDA-MB-453 and MCF7 cells. Western blot analysis confirmed induction of EGR1 protein at 1 h and ATF3 at 24 h. Paclitaxel, as well as digitoxin, inhibited the in vitro activity of the purified Na^+^-K^+^-ATPase; digitoxin enhanced the growth inhibitory effects of paclitaxel on Her2 overexpressing breast cancer cells.

**Conclusions:**

Our studies show the potential of digitoxin to prevent and treat breast cancer and indicate that the combination of digitoxin and paclitaxel is a promising treatment for ER negative breast cancer. These findings are the first to alert physicians to the possible dangers to patients who take a combination of digitoxin and paclitaxel. The potential dangers ensuing when paclitaxel and digitoxin are combined are dependent on the dose of digitoxin.

## BACKGROUND

Numerous studies have supported the notion that digitalis derivatives promise to be superior to existing adjuvant therapy as to effects and side-effects.[1,2]

**Figure 1:**
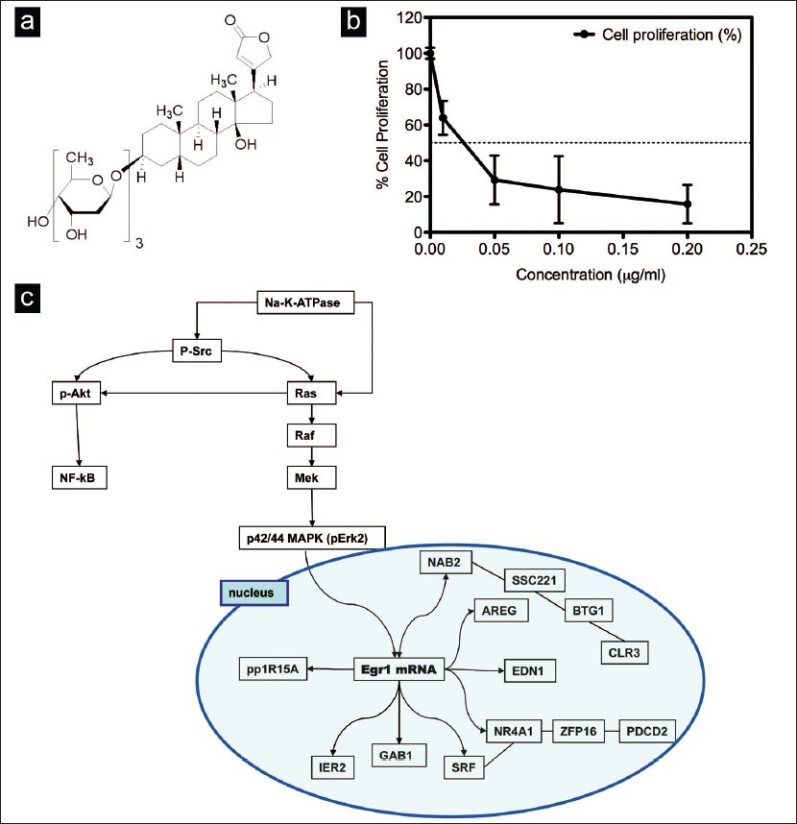
(a) Structure of digitoxin (Sigma, St. Louis, MO); (b) Effects of digitoxin on cell proliferation in MDA-MB-453 breast cancer cells, by the MTT assay. The DMSO control contained 0.33% DMSO. Similar results were obtained in an additional experiment. Bars: SD of triplicate assays. (c) Schematic diagram of genes altered after treating MDA-MB-453 cells with digitoxin at 1μg/ml for 6 h {adapted from STRING: Search Tool for Retrieving INteracting Genes/proteins (24-6)}.

*Digitalis purpurea* L. or common foxglove (family, Plantaginaceae) has been used for centuries as a folk remedy for heart ailments. More than thirty cardiac glycosides have been isolated from foxglove leaves, including digitoxin and digoxin. These compounds possess a steroid nucleus with a characteristic 17-carbon skeleton, an unsaturated lactone ring at the C17 position, and one or more glycosidic residues at C3 as seen in Figure 1a. The therapeutic range for digitoxin in the treatment of heart failure is narrow; it has a therapeutic plasma concentration greater than 10 ng/ml (13 nM), but is toxic at concentrations above 35 ng/ml (46 nM).

There is evidence that cardiac glycosides also have antitumor activity. Breast cancers from women on digitalis have more benign characteristics evidenced by a 9.6 times decrease in the recurrence rate after five years following a mastectomy.[3] However, a recent population-based case control study found a modest increase in breast cancer incidence among postmenopausal Danish women with any history of digoxin use.[4] The authors point out that the ability to affect the Na^+^-K^+^-ATPase appears to be dependent on the specific cardiac glycoside compound as well as the subunit set of the receiving Na^+^-K^+^-ATPase which is related to the tissue type.

Though it was initially thought that only toxic doses of digitoxin could be useful for anticancer activity, studies indicate that low doses of digitoxin induce apoptosis in malignant cell lines.[5,6] Crude extracts and several components present in foxglove demonstrate growth inhibition of serum-stimulated breast cancer cells. Digitoxin is 7.2 times more active than the aglycone on MCF7 human breast cancer cells.[3]

The growth inhibitory activity of cardiac glycosides may be related to their inhibition of the Na^+^-K^+^-ATPase, a member of evolutionarily conserved enzymes that couple ATP hydrolysis to ion translocation across cellular membranes.[7–9] The Na^+^-K^+^-ATPase contains two non-covalently linked alpha (catalytic) and beta subunits and a third subunit comprised of seven FXYD transmembrane proteins. When cardiac glycosides bind to the alpha subunit of the Na^+^-K^+^-ATPase, they potently inhibit the active transport of Na^+^ and K^+^ across cell membranes,[10] leading to a small increase in intracellular Na^+^ and resulting in a large increase in intracellular Ca^2+^. In heart muscle, this enhances the force of contraction.[2] Inhibition of the enzyme also releases and activates SRC, subsequently transactivating epidermal growth factor receptor, leading to assembly and activation of multiple signaling cascades such as Ras/Raf/ERK1/2 and phospholipase C/protein kinase C pathways and mitochondrial ROS production. Cardiotonic steroids have been reported to exert growth regulatory effects at nano- and sub-nanomolar concentrations that do not inhibit cellular Na^+^-K^+^-ATPase pumping activity.[6,11,12]

In animal models, digitoxin shows carcinogenesis inhibition on both two-stages of mouse skin papillomas induced by 7, 12-dimethylbenzanthracene (DMBA) and 12-O-tetradecanoylphorbol-13-acetate (TPA), and mouse pulmonary tumors induced by 4-nitroquinoline-N-oxide (4NQO) and glycerol.[13] The results of a recent study indicate that digitoxin (and related cardiac glycosides) can sensitize malignant prostate cancer cells to anoikis and thereby inhibit tumor metastasis.[14] This effect is due to inhibition of the Na^+^ /K^+^- pump which results in osmotic stress. In a mouse model, ouabain inhibited the growth of tumor metastases at safe doses and did not inhibit the growth of subcutaneous tumors.[14] These findings may partially explain the reports that digitoxin reduces metastases and clinical relapse in cancer patients. However, a recent report indicates that digitoxin inhibits protein synthesis resulting in cell death in both primary and cancer cell lines.[15] The effect appears to be due to inhibition of the Na^+^-K^+^--ATPase resulting in a decrease in K^+^, which is needed for protein synthesis. The findings raise concerns about dangers in ongoing clinical trials in humans.

Since the risks of digitoxin administration in humans are well known, it is appropriate to determine if doses less than or equivalent to those routinely used to treat patients with cardiac disorders are sufficient to achieve a chemopreventive or antitumor effect in patients with breast cancer. In the present study, we have used gene expression analysis to determine the molecular action of digitoxin on breast cancer cells and assessed digitoxin’s ability to synergize with the chemotherapy agent paclitaxel with respect to inhibition of cell proliferation. To determine whether digitoxin had similar effects on different breast cancer cell lines, we examined the effects on ER negative, Her2 overexpressing MDA-MB-453 cells and ER positive, Her2 low MCF7 cells.

## MATERIALS AND METHODS

All solvents and reagents were reagent grade; H_2_O was distilled and deionized. Digitoxin and paclitaxel were obtained from Sigma (St. Louis, MO). These agents were dissolved in dimethylsulfoxide (DMSO) (Sigma; St. Louis, MO) prior to addition to the cell cultures.

### Cell culture, proliferation assays and cell cycle analysis

MDA-MB-453 (ER^-^, Her2 overexpressing), MCF7 (ER^+^, Her2 low) and BT474 (ER^+^, Her2 overexpressing) cells were obtained from the ATCC (Manassas, VA). The above cells were grown in Dulbecco’s Modified Eagle’s medium (DMEM) (Gibco BRL Life Technologies, Inc., Rockville, MD) containing 10% (v/v) fetal bovine serum (FBS) (Gibco BRL) at 37 °C, 5% C0_2_. BT-474 cells were grown in DMEM plus 0.01 mg/ml bovine insulin. Every ATCC cell line comes with comprehensive authentication and quality assurance testing.

Cell proliferation was determined using: 1) the Coulter Counter assay, or 2) the MTT {3-(4,5-dimethyl-2-thiazol)-2,5-diphenyl-2H tetrazolilum bromide} (Dojindo; Tokyo, Japan) cell proliferation assay system, according to the manufacturer’s instructions (Roche Diagnostic, Mannheim, Germany). For the Coulter counter assay, cells were seeded at 2 × 10^4^ cells per well in 24 well plates (0.875 cm diameter) and allowed to attach for 24 h. The medium was replaced with fresh medium containing DMSO or digitoxin. The cells were treated for 96 h and the number was then determined using a Coulter Counter (Beckman Coulter Co., Fullerton, CA). For the MTT assay, cells were seeded at 1 × 10^4^ cells/well in 96-well plates and allowed to attach for 24 h. The medium was replaced with fresh medium containing DMSO or digitoxin. The cells were treated for 96 h after which the cells were incubated with MTT reagents and the absorbance read at 600 nM. Control and treated cells were compared using the student’s t-test (*P*<0.05). For cell cycle analysis, the cells were plated (3 × 10^5^) onto 6 cm dishes and allowed to attach for 24 h. Then the medium was replaced with DMEM containing DMSO or digitoxin. After 24 h, the cells were analyzed by DNA flow cytometry, as described previously.[16]

### Calculating the combination index

To determine the combination index (CI), we exposed MDA-MB-453 cells to all combinations of four concentrations of each of the agents tested and a solvent control. The results of the MTT assay were analyzed for possible synergistic effects using the median effect principle.[17] We employed variable ratios of drugs and assumed mutually exclusive equations.[18]

### Enzymatic assay of adenosine 5’-triphosphatase

The enzymatic assay of ATPase (adenosine 5’-triphosphatase, EC 3.6.1.3) followed the Sigma Prod. No. A-7510 protocol (Sigma-Aldrich, St. Louis, MO, USA). Digitoxin or paclitaxel was added to ATPase (0.05 ml, 0.5 unit/ml, Sigma-Aldrich, St. Louis, MO, USA), mixed and equilibrated for 5 min at 37°C. [P] was determined by the Taussky-Shorr method.

### RNAi-mediated gene knockdown

To test the functional relevance of ERK2 (p42/44MAPK pathway), we examined the growth inhibitory effects of digitoxin on MDA-MB-453 cells using the model system RNAi-mediated gene knockdown.[19] We pretreated cells with siRNA to ERK2 (Hs/Mm MAPK1 siRNA) or non-silencing RNA (Qiagen, Valencia, CA) for 24 h, then treated with digitoxin at 0.4 *μ*g/ml for 24 h and assayed the percent surviving cells. Cells that were pretreated with siRNA and then treated with digitoxin were also used to prepare extracts for Western blot analysis to confirm the ERK2 knockdown.

### RNA isolation and oligonucleotide microarray analysis

We treated MDA-MB-453 cells with 20 ng/ml, 0.1 (26 nM), 0.2 or 1 *μ*g/ml of digitoxin for 6 and 24 h and MCF7 cells with digitoxin at 1 *μ*g/ml for 6 and 24 h. Total cellular RNA was extracted using Trizol (Invitrogen; Carlsbad, CA) according to the manufacture’s protocol with minor modifications, and then purified twice with Qiagen’s RNeasy column, as previously described. Total RNA (8 *μ*g) was reverse transcribed with T7-(dT)24 primer and Super Script III reverse transcriptase (Invitrogen). After purification, cDNA was *in vitro* transcribed into biotin labeled antisense cRNA with the BioArray high yield RNA transcript labeling kit (Enzo Life Sciences; Farmingdale, NY), according to a modified Affymetrix protocol. cRNA (15 *μ*g) was fragmented into the final probe and hybridized to human U133A 2.0 gene chips (Affymetrix, Inc.; Santa Clara, CA), comprised of more than 22,000 probe sets.

### Real-time quantitative RT-PCR and Western blot analysis

We performed real-time RT-PCR analysis on two technical replicates of at least two biological sample replicates.[19] Primer sequences used in qPCR are listed in Table 1. For Western blot analysis, cells were treated for increasing periods with approximately the IC_50_, twice the IC_50_ or ten times the IC_50_ concentration, measured at 48 h, of digitoxin. Western blot analysis was performed as described previously.[16] The membrane was incubated with the primary antibodies to ATF3 and EGR1 (Santa Cruz Biotechnology, Santa Cruz, CA); β-actin (Santa Cruz Biotechnology, Santa Cruz, CA) was used as a loading control.

**Table 1:** Designed primer sequence used in RT-PCR

Symbol	Forward sequence	Reverse sequence
GAPD	ggcctccaaggagtaagacc	aggggtctacatggcaactg
ATF3	tgggaggactccagaagatg	gacagctctccaatggcttc
EGR1	gagaaggtgctggtggagac	tgggttggtcatgctcacta
GDF15	ctccgaagactccagattcc	agagatacgcaggtgcaggt
CDKN1A	gcctggactgttttctctcg	attcagcattgtgggaggag
HSF2	atgggaaccctgcttcttct	ttgggttggttctgggtcta
DNAJB4	ccggacaagaacaaatctcc	cctcctttcaacccttcctc
HMGCR	gacctttccagagcaagcac	agctgacgtacccctgacat
HMGCS1	ccccagtgtggtaaaattgg	tggcctggacttaacattcc
INSIG1	gacagtcacctcggagaacc	caccaaaggcccaaagatag
ATF4	ccaacaacagcaaggaggat	gtgtcatccaacgtggtcag
GADD34	ggaggctgaagacagtggag	cctctagggacactggttgc
CDC16	cgatggctgcttacttcaca	cagagcttggctgaagaacc
ATP1A1	tgtgattctggctgagaacg	tgctcataggtccactgctg

In Table 1, primers were designed using Primer3 software from the Massachusetts Institute of Technology (frodo.wi.mit.edu/cgi-bin/primer3/primer3_www.cgi).

### Gene expression analysis

We treated MDA-MB-453 breast cancer cells and collected RNA for gene expression analysis, as described above. We used microarray analysis and an unbiased informatics approach to find the genes and signaling pathways whose expression was altered by exposure of the cells to digitoxin. Two or three replicates of each microarray were performed to determine intra-sample variation. To augment the analysis, we examined the effects of four doses for two time periods.

All analyses were performed using the AffyLimma GUI package in the open-source Bioconductor suite. All samples were normalized to remove chip-dependent regularities using the GCRMA method of Irizarry *et al*.[20] The statistical significance of differential expression was calculated using the empirical Bayesian LIMMA (LI Model for MicroArrays) method of Smyth *et al*.[21] A cut-off *B*>0 was used for the statistical significance of gene expression. The manuscript reports variability in terms of a *P*-value representing the probability that differences between treated and untreated could occur by chance. The *P*-value accounts for both the variability within groups (in this case, the treatment groups and control) and the variability between groups.

The genes that displayed significant changes in levels of expression were assigned to Gene Ontology categories and KEGG Pathways.[22] Intersections between treatments were calculated using the Gene Traffic program. Clustering was performed with the Program Cluster 3.0.[23]

## RESULTS

### The effects of digitoxin on breast cancer cell growth

After treating Her2 overexpressing, ER low MDA-MB-453 breast cancer cells with increasing concentrations of digitoxin for 96 h, we assessed digitoxin’s effects by the MTT assay. The concentration of digitoxin that caused 50% inhibition of cell proliferation, the IC_50_ value, was about 0.025 *μ*g/ml (0.04 *μ*M) [Figure 1b]. The Coulter counter assay indicated that digitoxin inhibited growth of the MDA-MB-453 and Her2 overexpressing, ER^+^ BT474 breast cancer cells, with IC_50_ values of 0.04 *μ*g/ml (0.05 *μ*M) and 0.03 *μ*g/ml (0.04 *μ*M), respectively. The IC_50_ values of 0.025 to 0.03 *μ*g/ml are within the therapeutic range, 10-35 ng/ml (13-46 nM) for digitoxin. Digitoxin was less potent on ER positive, Her2 low MCF7 breast cancer cells, with an IC_50_ value of 0.2 *μ*g/ml.

### Effects of digitoxin on cell cycle distribution in MDA-MB-453 human breast cancer cells

Effects on cell cycle distribution at 24 h after exposing MDA-MB-453 cells to 0, 0.2 (0.26 *μ*M) or 2 *μ*g/ml are shown in Table 2. After treatment with digitoxin at 0.2 or 2 *μ*g/ml, there was an increase in the subG1 peak, which may indicate apoptosis. Digitoxin induced a dose-dependent increase in the percent of cells in G2 and a decrease in the percent of cells in G1, and, at the higher dose, decreases in G1 and S phases.

**Table 2:** Effect of digitoxin on cell cycle distribution in MDA-MB-453 cells

Treatment	SubG1		G1		S		G2	
0 μg/ml	1.93	(1.02)	57.75	(1.20)	26.90	(0.99)	11.45	(4.74)
0.2 μg/ml	6.77	(1.06)	46.50	(1.98)	27.65	(0.64)	19.05	(0.07)
2.0 μg/ml	5.78	(1.02)	41.90	(2.26)	17.80	(0.42)	32.10	(1.27)

Table 2. MDA-MB-453 cells were treated with 0.2 or 2 μg/ml of digitoxin and analyzed at 48 h by DNA flow cytometry. The values indicate the percent of cells in the indicated phases of the cell cycle. The control contained 0.01% DMSO. Standard deviations are indicated in parentheses..

### Alterations in gene expression induced by a nontoxic dose of digitoxin

When we examined the effect of a nontoxic dose of digitoxin (20 ng/ml) on gene expression patterns at 24 h, we found that digitoxin significantly altered the expression of 22 genes. The 11 upregulated genes included corneodesmosin (CDSN), keratin 23 (histone deacetylase inducible) (KRT23), desmoplakin (DSP), and cysteine-rich secretory protein LCCL domain containing 2 (CRISPLD2); the 11 downregulated genes included calmegin, chromosome 9 open reading frame 127 (C9orf127), ubiquitin specific protease 34 (USP34) and baculoviral IAP repeat-containing 1(BIRC1) [Table 3].

**Table 3:** Differentially expressed genes after treating MDA-MB-453 cells with digitoxin at 20 ng/mL for 24 h, B>0

Category	ID	Symbol	Name	M	*P* value	B
Apoptosis	206192_at	CDSN	corneodesmosin	2.255	1.34E-05	4.961
	218963_s_at	KRT23	keratin 23 (histone deacetylase inducible)	2.231		1.034
	204890_s_at	BIRC1	baculoviral IAP repeat-containing 1	-0.546		0.524
Stress	204635_at	RPS6KA5	ribosomal protein S6 kinase, 90 kDa, polypeptide 5	-0.869	0.0215	2.574
Protein	200606_at	DSP	desmoplakin	1.176		0.709
	221541_at	CRISPLD2	cysteine-rich secretory protein LCCL domain containing 2	0.842		0.706
	215339_at	NKTR	natural killer-tumor recognition sequence	-0.418		1.261
	212980_at	USP34	ubiquitin specific protease 34	-1.098		0.388
	205830_at	CLGN	calmegin	-1.469		0.297
Transcription	207839_s_at	C9orf127	chromosome 9 open reading frame 127	-1.117	1.34E-05	4.914
	221810_at	RAB15	RAB15, member RAS oncogene family	0.403		0.632
Ion	203402_at	KCNAB2	potassium voltage-gated channel, shaker-related subfamily, beta member 2	0.383		0.486
	210486_at	ANKMY1	ankyrin repeat and MYND domain containing 1	0.199		1.571
Signal transduction	210222_s_at	RTN1	reticulon 1	0.172		0.663
Nucleotide	201766_at	ELAC2	elaC homolog 2 (E. coli)	-0.158		0.289
	212913_at	MSH5	mutS homolog 5 (E. coli)	-0.637		0.156
Function unknown	205796_at	FLJ11336	NA	-0.188		0.925
	222307_at	LOC282997	NA	-0.342	0.447
	215364_s_at	KIAA0467	NA	-0.416	0.0122	3.049
	219054_at	FLJ14054	NA	1.105		1.384
	221843_s_at	KIAA1609	NA	0.916	0.015	2.839

Table 3. Exponentially dividing cultures of MDA-MB-453 cells were treated with digitoxin at 20 ng/ml and then collected for RNA extraction at 24 hours. Microarray analysis was performed as described in Materials and Methods. Fold-change (log) is the mean of the ratio of hybridization signals in digitoxin treated versus DMSO control treated cells. NA designates function not known. B>0, *P*<0.05, unless otherwise noted.

It is noteworthy that several of these genes are activated by SRC mediated pathways. RPS6KA5 is activated by ERK. RAB15 is a member of the RAS oncogene family and involved in GTP binding. BIRC1 is an anti-apoptotic gene and C9orf127 is involved in regulating the cell cycle. KCNAB2 has a role in mediating potassium voltage-gated channels. Of the 22 genes, four were also downregulated after treatment with a five-fold higher dose. These included genes that were activated by ERK (RPS6KA5) or that mediate apoptosis (BIRC1), cell cycle regulation (C9orf127), or ubiquitin-dependent protein catabolic process (USP34).

### Alterations in Gene Expression Induced by Various Treatments with Digitoxin

Since only 22 genes were altered after treatment with the nontoxic dose at 24 h, we treated MDA-MB-453 breast cancer cells with digitoxin at three higher concentrations, 0.1 (0.13*μ*M), 0.2 or 1 *μ*g/ml in order to maximize cellular responses to digitoxin and reveal its mechanism of action. The number of genes impacted by the individual treatments with digitoxin (|M| >0, *P*<0.05) increased in a dose- and time-dependent manner. Thus, treatment with 0.1 *μ*g/ml for 6 and 24 h altered two or eight genes, respectively; 0.2 *μ*g/ml for 6 and 24 h altered six or 88 genes, respectively; 1 *μ*g/ml for 6 and 24 h altered 87 or 1491 genes, respectively. Under all treatment conditions at 6 h (B>0, *P*<0.05, |M| >0), more genes were induced than repressed by a factor of about 1.0 to 2.5-fold, but the inverse (0.5-0.7-fold) was true at 24 h.

Using the program Gene Traffic to identify commonly perturbed genes amongst the three doses of digitoxin and two time periods, we found no commonly perturbed genes at 0.1 *μ*g/ml for 6 or 24 h, two commonly perturbed genes at 0.2 *μ*g/ml, and 61 genes or 61/87 genes (at 6 h) at 1 *μ*g/ml. Thus, the lower doses of digitoxin altered different sets of genes at 6 and 24 h, while the highest dose altered similar sets of genes at the two time points.

Affymetrix NetAffx analysis showed a significant effect on stress response genes after treatment with the highest dose of digitoxin, 1 *μ*g/ml, for 6 h [Table 4]. Among the early effects were upregulation of stress (EGR1, NAB2), apoptotic (IHPK2, ARID5B), lipid biosynthetic (SC5DL), transcription regulation (NR4A1, ZNF297B, RORA), anti-proliferation (BTG1) and RNA processing (DDX26) genes and downregulation of cell cycle (C10orf7), replication (POLR3B) and transcription (EIF2B1) genes. As predicted,[5] digitoxin altered the response of genes involved in calcium metabolism (IHPK2, NR4A1). The analysis program STRING: Search Tool for Retrieving INteracting Genes/proteins revealed that EGR1 is at the hub of genes induced by digitoxin; EGR1 appears to induce the expression of NAB2, which mediates the feedback inhibition of EGR1, IER2 and ppp1R15A [Figure 1c].[24–26]

**Table 4:** Differentially expressed genes after treating MDA-MB-453 cells with digitoxin at 1.0 μg/ml for 6 h

Category	ID	Symbol	Name	M	*P* value
Transcription	36711_at	MAFF	v-maf musculoaponeurotic fibroscarcoma oncogene homolog F (avian)	8.68	0.00805
	201693_s_at	EGR1	early growth response 1	6.25	0.00028
	216017_s_at	NAB2	NGFI-A binding protein 2 (EGR1 binding protein 2)	5.75	0.00022
	205193_at	MAFF	v-maf musculoaponeurotic fibroscarcoma oncogene homolog F (avian)	5.16	0.00103
	202340_x_at	NR4A1	nuclear receptor subfamily 4, group A, member 1	3.35	0.00269
	201725_at	C10orf7	chromosome 10 open reading frame 7	−1.24	0.00712
	214185_at	KHDRBS1	KH domain containing, RNA binding, signal transduction associated 1	2.22	0.00366
DNA binding	210426_x_at	RORA	RAR−related orphan receptor A	0.679	0.00100
	212614_at	ARID5B	AT rich interactive domain 5B (MRF1-like)	1.24	0.00330
	219459_at	POLR3B	polymerase (RNA) III (DNA directed) polypeptide B	−1.92	0.00360
Protein binding	203002_at	AMOTL2	angiomotin like 2	2.84	0.00266
	204182_s_at	ZNF297B	zinc finger protein 297B	2.58	4.53E-06
	221890_at	ZNF335	zinc finger protein 335	2.47	0.00767
	78330_at	ZNF335	zinc finger protein 335	0.62	0.00393
	201823_s_at	RNF14	ring finger protein 14	−1.13	0.00784
	209630_s_at	FBXW2	F-box and WD-40 domain protein 2	−1.93	0.00777
	218819_at	DDX26	DEAD/H (Asp-Glu-Ala-Asp/His) box polypeptide 26	1.39	0.00504
Cell growth (-), apoptosis (+), oxidative stress	218192_at	IHPK2	inositol hexaphosphate kinase 2	2.83	0.00379
	200920_s_at	BTG1	B-cell translocation gene 1, anti-proliferative	1.72	0.00121
	200797_s_at	MCL1	myeloid cell leukemia sequence 1 (BCL2-related)	1.86	0.00378
	214056_at	MCL1	myeloid cell leukemia sequence 1 (BCL2-related)	1.76	0.00634
Phase 2	221906_at	TXNRD3	thioredoxin reductase 3	−0.76	0.00626
Protein metabolism	210592_s_at	SAT	spermidine/ spermine N1-acetyltransferase	2.66	0.00478
	202140_s_at	CLK3	CDC-like kinase 3	1.45	0.00155
	201632_at	EIF2B1	eukaryotic translation initiation factor 2B, subunit 1 alpha, 26 kDa	−1.21	0.00449
Lipid metabolism, biosynthesis	211423_s_at	SC5DL	sterol-C5-desaturase (ERG3 delta-5-desaturease homolog, fungal)-like	1.98	0.00453
Function unknown	215150_at	DKFZp451J1719	NA	2.75	0.00592
	219397_at	FLJ13448	NA	1.59	0.00557
	219016_at	FLJ13149	NA	1.30	0.00015

Table 4 Exponentially dividing cultures of MDA-MB-453 cells were treated with digitoxin at 1.0 μg/ml and then collected for RNA extraction at 6 hours. Microarray analysis was performed as described in Materials and Methods. Fold-change (log) is the mean of the ratio of hybridization signals in digitoxin treated versus DMSO control treated cells. NA designates function not known. B>0, M>3, *P*<0.05, unless otherwise noted.

In the cells treated with digitoxin at 0.2 *μ*g/ml, the three main genes that were significantly altered and possibly contribute to cancerous behavior were NAB2, ARID5B, and MCL1. SC5DL and EGR1 appear with *P* values slightly higher than 0.05, indicating their involvement as well. It appears that these are among the primary genes affected by digitoxin.

### Hierarchical clustering of alterations in gene expression after treating cells with digitoxin

**Figure 2:**
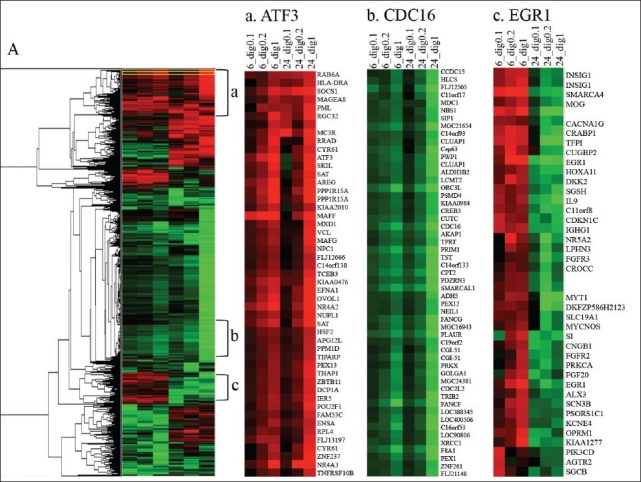
Hierarchical clustering of differentially expressed genes analyzed on U133A 2.0 Affymetrix chips after treating MDA-MB-453 cells with digitoxin at 0.1, 0.2 or 1.0 μg/ml for 6 and 24 h. Clustering was performed with the Program Cluster 3.0 (23). We restricted probesets to those that corresponded to an absolute value of M (log fold) > 2.0 for at least one of the conditions. The threshold for color in the hierarchical clustering map is M > 3 log fold. Fold change indicates relative expression in digitoxin versus DMSO control cells. To pick the blowup region, the area containing a specific gene was expanded to include a well-defined expression pattern. A) the full hierarchical clustering map, which contains 4706 probesets (a) upregulated gene region amplified for ATF3; (b) down regulated gene region amplified for CDC16; (c) upregulated gene region amplified for EGR1; red, upregulated; green, downregulated.

We used hierarchical clustering to reveal genes that are coordinately controlled [Figure 2]. Figure 2a shows the full hierarchical clustering map, which contains 4706 probe sets. Figure 2 (a, b and c) are expanded displays of specific subcategories of these probe sets. Figure 2a contains a cluster of genes, which, like ATF3, were mainly activated after treatment with digitoxin at 1 *μ*g/ml, for either 6 or 24 h. However, some of these genes were also activated after treatment with 0.1 or 0.2 *μ*g/ml, for 6 or 24 h, including additional stress response genes (GADD34, IER2, and HSF2). Figure 2b displays genes that were downregulated by treatment with digitoxin such as the cell cycle control gene CDC16 and replication gene ORC3, which were repressed after treatment with digitoxin at 1 *μ*g/ml for 6h, further repressed at 24 h, and slightly repressed after treatment with digitoxin at 0.2 *μ*g/ml for 6 or 24 h. The cluster of genes displayed in Figure 2c contains the gene EGR1 which was upregulated after treatment with 0.1, 0.2 or 1 *μ*g/ml of digitoxin for 6 h, but not evident at 24 h. This cluster also contained lipid biosynthetic genes (INSIG1). A fourth region, expanded for CDKN1A, showed a progressive increase in expression after treatment with digitoxin at 0.1, 0.2 or 1 *μ*g/ml for 6 or 24 h, with a more pronounced increase at 24 h (data not shown). This cluster also contained the stress gene DNAJB4 and the apoptotic gene GDF15.

### The effects of digitoxin on expression of specific mRNAs determined by real-time RT-PCR

**Figure 3:**
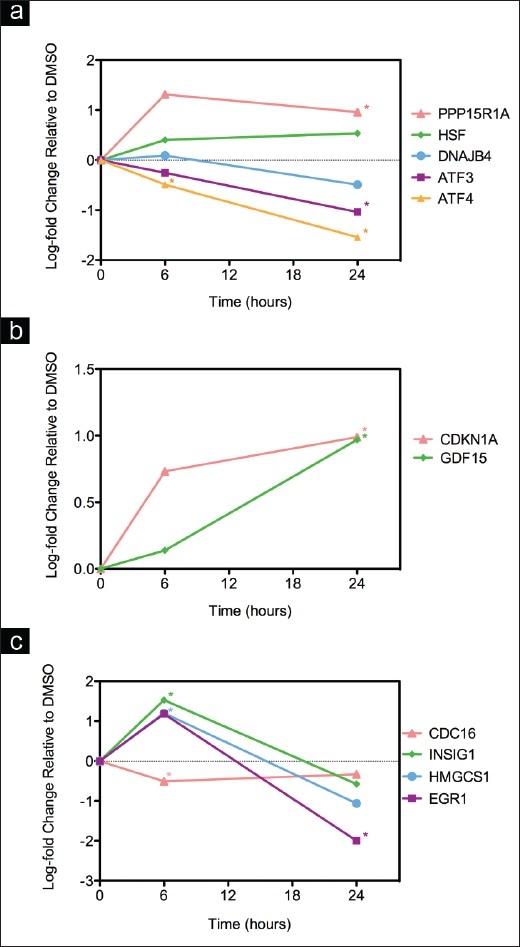
Real-time RT-PCR analysis: a, b, c) after treating MDAMB-453 cells with digitoxin at 0.1 *μ*g/ml for 6 and 24 h. The cells were treated with 0.1 μg/ml of digitoxin and, after 6 and 24 h, extracts were prepared and analyzed by real-time RT-PCR, as described in Materials and Methods; a, b and c display different patterns of gene expression.* indicates *P*<0.05; Fold change indicates relative expression in digitoxin versus DMSO control cells.

To verify some of the digitoxin-induced changes that we observed in gene expression detected by microarray analysis, we performed real-time RT-PCR analysis on the same RNA samples. We examined 12 genes related to the stress response or cell cycle control. The RT-PCR results [Figure 3 and Table 5a, b] were consistent with the microarray analysis results. To show how the data varied, *P*-values are indicated for all microarray and PCR genes in the Tables.

Consistent with the hierarchical clustering results, there were four main patterns of expression: (1) mRNAs for the ER stress gene EGR1 and the lipid genes INSIG1 and HMGCS1 increased at 6 h and decreased at 24 h [Figure 3c]; (2) the integrated stress response (ISR) genes ATF3, ATF4, PPP1R15A and DNAJB4 and HSF2 displayed different expression patterns after treatment with the three doses; at 0.1 *μ*g/ml the expression of ATF3, ATF4 and DNAJB4 decreased from 6 to 24 h and the expression of HSF2 and PPP1R15A increased and then leveled off [Figure 3a], whereas after treatment with 1 *μ*g/ml, they showed a progressive increase in mRNA expression; (3) expression of the apoptotic genes GDF15-A, CDKN1A and the Na^+^-K^+^-ATPase ATP1A1 progressively increased after treatment with digitoxin at all doses for 6 or 24 h [Figure 3b, Table 5a, b]; and (4) the expression of CDC16 decreased at 6 h and then leveled off [Figure 3c]. Thus, digitoxin induced cholesterol and ISR genes in a time and dose dependent fashion and progressively induced the expression of apoptotic genes.

**Table 5:** Comparison of the effects of digitoxin on selected genes by real-time PCR and microarray analysis

A. 6 hours														
Category	Gene	Affymetric number	Fold-change relative to DMSO fold change (*P* value)											
			Digitoxin treament (6h, 0.1 μg/mL)				Digitoxin treatment (6h, 0.2 μg/mL)				Digitoxin treatment (6h, 1.0 μg/mL)			
			RT-PCR		Microarray		RT-PCR		Microarray		RT-PCR		Microarray	
Stress response	HSF2	211220_s_at	0.41	(0.059)	1.27	(1.00)	0.91	*	1.93	(0.35)	1.34	*	2.45	(0.05)
	ATP1A1	220948-s_at	0.19	(0.170)	0.16	(1.00)	0.62	(0.088)	0.22	(1.00)	0.42	(0.03)	0.24	(1.00)
	ATF3	202672_s_at	−0.25	(0.380)	0.16	(1.00)	1.15	(0.002)	1.66	(1.00)	3.99	*	6.91	(0.10)
	DNAJB4	202887_s_at	0.09	(0.480)	0.80	(1.00)	0.30	(0.003)	0.88	(1.00)	1.43	*	2.52	(1.00)
	EGR-1	211936_at	1.18	(0.130)	1.68	(1.00)	3.26	(0.002)	3.43	(1.00)	5.15	*	5.05	(1.00)
	INSIG1	201625_s_at	1.53	(0.002)	1.71	(1.00)	2.29	*	2.33	(1.00)	2.77	*	2.85	(0.88)
	ATF4	200779_at	−0.49	(0.003)	−0.38	(1.00)	−0.12	(0.260)	−0.19	(1.00)	0.97	*	0.56	(1.00)
	PPP1R15A	37028_at	1.32	(0.065)	1.78	(1.00)	2.48	*	3.42	*	4.05	*	5.49	(0.02)
Promote apoptosis	CDKN1A	209383_at	0.73	(0.089)	1.22	(1.00)	1.62	*	2.06	(1.00)	2.61	*	3.41	(0.34)
	GDF15	221577_x_at	0.14	(0.680)	0.19	(1.00)	0.60	(0.120)	0.73	(1.00)	1.34	*	1.75	(1.00)
Cell cycle	CDC16	209658_at	−0.51	(0.007)	−0.83	(1.00)	−0.67	(0.001)	−1.55	(1.00)	−1.31	*	−2.82	(0.05)
Cholesterol/fatty acid biosynthesis	HMGCS1	205822_s_at	1.20	(0.011)	1.09	(1.00)	2.04	*	1.69	(1.00)	2.75	*	2.53	(1.00)

**B. 24 hours**														
**Category**	**Gene**	**Affymetric number**	**Fold-change relative to DMSO fold change (P.value)**											
			**Digitoxin treament (24h, 0.1 μg/mL)**				**Digitoxin treatment (24h, 0.2 μg/mL)**				**Digitoxin treatment (24h, 1.0 μg/mL)**			
			**RT-PCR**		**Microarray**		**RT-PCR**		**Microarray**		**RT-PCR**		**Microarray**	
Stress response	HSF2	211220_s_at	0.40	(0.062)	1.17	(0.54)	0.63	(0.01)	1.64	(1.00)	2.32	*	3.23	(0.01)
	ATP1A1	220948-s_at	−0.10	(0.380)	0.35	(1.00)	0.00	(0.98)	0.5	(1.00)	−0.03	(0.780)	0.67	(1.00)
	ATF3	202672_s_at	−1.04	(0.006)	−0.10	(1.00)	0.62	(0.13)	0.85	(1.00)	5.32	*	8.42	(0.02)
	DNAJB4	202887_s_at	−0.49	(0.150)	0.20	(1.00)	0.35	(0.18)	2.15	(1.00)	3.51	*	6.00	*
	EGR-1	211936_at	−2.00	*	−2.40	(1.00)	−3.38	*	−4.07	(1.00)	−2.30	*	−2.49	(1.00)
	INSIG1	201625_s_at	−0.58	(0.150)	−0.50	(1.00)	−1.51	*	−1.31	(1.00)	−1.08	(0.015)	−061	(1.00)
	ATF4	200779_at	−1.54	*	−1.08	(1.00)	−0.66	(0.03)	−0.49	(1.00)	2.71	*	1.65	(1.00)
	PPP1R15A	37028_at	0.96	(0.041)	0.69	(1.00)	1.64	*	1.80	*	4.36	*	5.50	(0.02)
Promote apoptosis	CDKN1A	209383_at	0.99	(0.021)	1.52	(1.00)	1.62	*	2.76	(1.00)	2.61	*	5.22	(0.01)
	GDF15	221577_x_at	0.97	(0.010)	1.41	(1.00)	2.35	(0.01)	2.94	(1.00)	3.20	*	4.25	(1.00)
Cell cycle	CDC16	209658_at	−0.33	(0.120)	−0.87	(1.00)	−.044	(0.19)	−1.71	(1.00)	−1.58	(0.001)	−4.93	*
Cholesterol/fatty acid biosynthesis	HMGCS1	205822_s_at	−1.06	(0.071)	−0.54	(1.00)	−1.89	*	−1.88	(1.00)	−2.01	(0.007)	−1.01	(1.00)

Table 5 A) and B). Exponentially dividing cultures of MDA-MB-453 cells were treated with digitoxin at 0.1, 0.2 or 1.0 μg/ml, and then collected for RNA extraction at 6 and 24 hours. Microarray analysis was performed as described in Materials and Methods. Fold-change (log) is the mean of the ratio of hybridization signals in digitoxin treated versus DMSO control treated cells. Real-time RT-PCR was performed as previously described [19];

* *P* values are < 0.00 I, unless indicated in parentheses.

### The effects of digitoxin on expression of ATF3 and EGR1 protein in MDA-MB-453 cells

**Figure 4:**
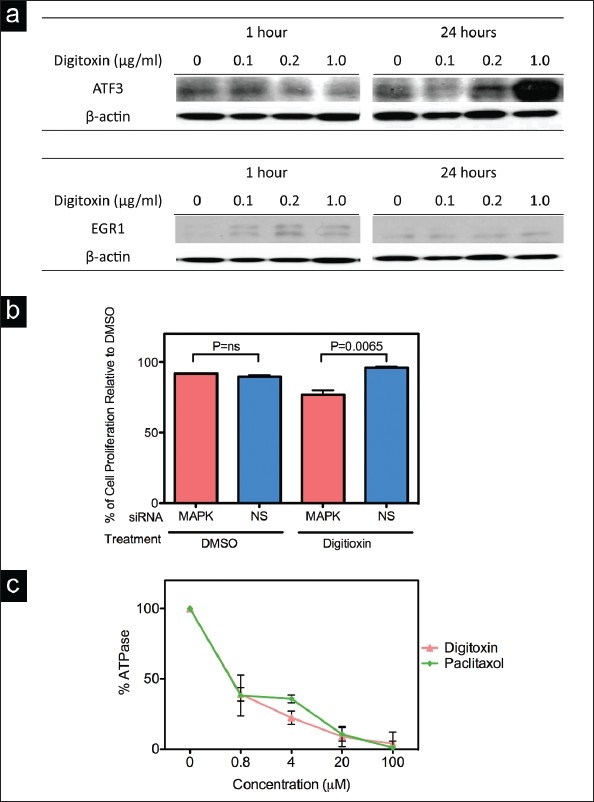
a) Effects of digitoxin on the level of ATF3, EGR1 protein. MDA-MB-453 cells were treated with 0, 0.1, 0.2 or 1 *μ*g/ml of digitoxin and after 1 and 24 h extracts were prepared and analyzed by Western blotting; an antibody to β-actin was used as a loading control. b) siRNA to ERK2. Cell were pretreated with control (nonsilencing) or MAPK1 (ERK2) siRNA for 24 h, exposed to digitoxin (0.4 μg/ml) for 24 h and percent inhibition of cell proliferation determined by the MTT assay. Percentages are normalized to DMSO. c) Inhibition of Na+-K+-ATPase activity in response to increasing concentrations of paclitaxel or digitoxin. The Na+-K+-ATPase assay was performed as described in Materials and Methods. The DMSO controls contained 3.3% DMSO. BARS: SD of triplicate assays.

Digitoxin significantly upregulated the expression of the transcription factor ATF3 and the early response gene EGR1, by both microarray and RT-PCR analysis. Western blot analysis confirmed that when MDA-MB-453 cells were treated with digitoxin, EGR1 protein was induced after treating with digitoxin at 0.1, 0.2 or 1.0 *μ*g/ml for 1 h, whereas the ATF3 protein was induced after treating with digitoxin at 1.0 *μ*g/ml for 24 h [Figure 4a].

### RNAi-mediated gene knockdown

To confirm an effect on MAPK signaling, we examined the growth inhibitory effects of digitoxin using the model system RNAi-mediated gene knockdown. Pretreating cells with siRNA to MAPK1 (ERK2) before treating with digitoxin (0.4 *μ*g/ml) for 24 h resulted in decreased cell proliferation (96.0% to 76.9%), indicating that MAPK1 is involved in the survival phase of the digitoxin-induced stress response [Figure 4b]. Western blot analysis confirmed that pretreatment with ERK2 siRNA did, in fact, reduce the expression of ERK2 protein in MDA-MB-453 cells by about 0.58 (MAPK1: NS siRNA).

### Gene expression analysis of the effects of digitoxin on MCF7 cells

**Figure 5:**
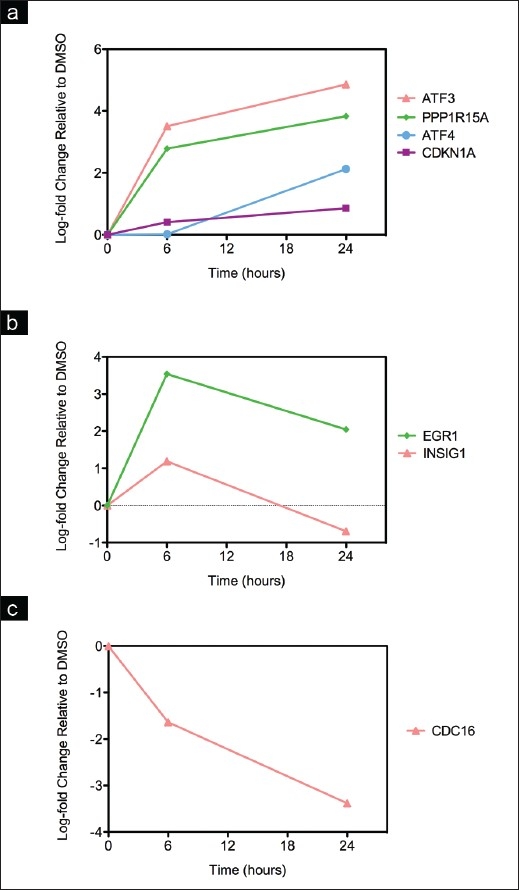
Real-time RT-PCR analysis after treating MCF7 cells with digitoxin at 1 *μ*g/ml for 6 and 24 h, as described in Materials and Methods. The cells were treated with 1.0 μg/ml of digitoxin and, after 6 and 24 h, extracts were prepared and analyzed by real-time RT-PCR, as described in Materials and Methods. a, b and c display different patterns of gene expression. All *P* values were <0.05, except ATF4 at 6h; Fold change indicates relative expression in digitoxin versus DMSO control cells.

To determine whether other breast cancer cell lines would react similarly to digitoxin, we tested the effect of digitoxin at 1 *μ*g/ml for 6 or 24 h on ER positive MCF7 cells, using real-time RT-PCR analysis. We observed three patterns of expression similar to those found with MDA-MB-453 cells. Thus, mRNAs for: 1) the ER stress gene EGR1 and the lipid gene INSIG1 increased at 6 h and decreased at 24 h; 2) the stress genes ATF4, ATF3 and GADD34 showed a progressive increase in expression of the related mRNAs after treatment with digitoxin at 1 *μ*g/ml for 6 or 24 h; and 3) the cell cycle gene CDC 16 showed a progressive decrease in expression [Figure 5a–c].

### Digitoxin in combination therapy

To find an effective combination treatment, we tested the effects of paclitaxel, a common treatment for breast cancer, on the *in vitro* activity of the purified Na^+^-K^+^-ATPase. The respective IC_50_ values for digitoxin and paclitaxel were 0.62 and 0.61 *μ*M, respectively [Figure 4c]. In view of these effects, we explored the effects of digitoxin in combination with paclitaxel on cell proliferation of MDA-MB-453 cells.

**Figure 6:**
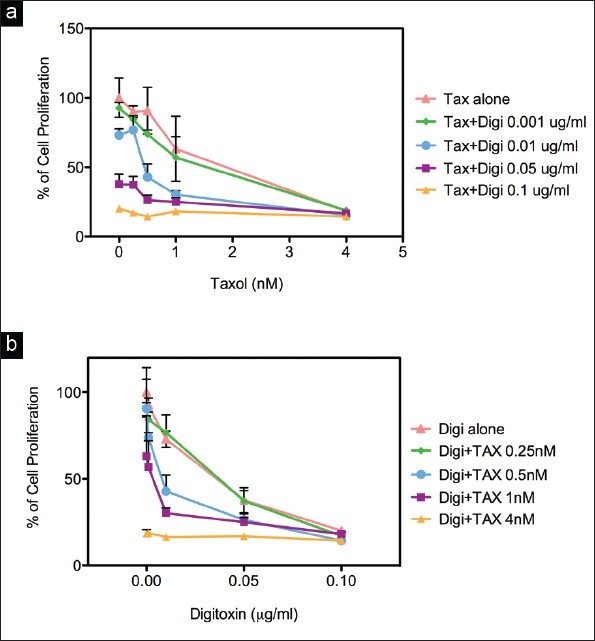
Effects of digitoxin alone or in combination with paclitaxel (TAX) on cell proliferation in MDA-MB-453 breast cancer cells: a) x-axis, TAX concentration, b) x-axis, digitoxin concentration. The DMSO control contained < 0.1% DMSO; Bars: SD.

Resulting data from combined increases of both paclitaxel (TAX) and digitoxin are shown in Figure 6. In these studies, the two test agents were added simultaneously to the cells. The data were analyzed for the respective combination indices (CI) [Figure 6a, b; Table 6]. An additive effect was seen with as little as 0.01 *μ*g/ml digitoxin and 0.5 nM of paclitaxel, and moderate synergy with 0.01 *μ*g/ml digitoxin and 1 nM of paclitaxel. With the former combination (0.01 *μ*g/ml digitoxin, 0.5 nM paclitaxel), the percent viable cells decreased from 90.6% after treatment with paclitaxel alone to 42.9% after combination treatment, *P*<0.01 (digitoxin alone: 73.0%). Addition of digitoxin (0.01 *μ*g/ml) to paclitaxel (1 nM) decreased cell survival from 63.2% to 30.3% (*P*< 0.01).

**Table 6:** Combination index (CI) values for MDA-MD-453 cells treated with digitoxin plus paclitaxel

	Digitoxin (ug/ml)								
		0.001		0.01		0.05		0.1	
TAX (nM)	0.25	4.18	(- -)	2.79	(- -)	2.23	(- -)	2.23	(- -)
	0.5	2.42	(- -)	1.04	(+/-)	0.47	(+++)	0.47	(+++)
	1	2.15	(- -)	0.76 (++)	0.20	(+++)	0.20	(+++)
	4	1.95	(- -)	0.57	(+++)	0	(+++)	0	(+++)
Symbols:	CI
- -				>1.3			Antagonism		
-				1.1-1.3			Moderate antagonism		
+/-				0.9-1.1			Additive effect		
+				0.8-0.9			Slight synergism		
+ +				0.6-0.8			Moderate synergism		
+ + +				<0.6			Synergism		

Table 6. IC_50_ values determined from the graphs in Figure 5 were used to obtain combination index values: CI = {IC_50_ (digitoxin + paclitaxel) / IC_50_ (digitoxin alone)} + {IC_50_ (paclitaxel + digitoxin) / IC_50_ (paclitaxel alone)}.

## DISCUSSION

Ascertaining 1) the effectiveness of digitoxin at nontoxic doses, 2) its mechanism of action, and 3) its ability to synergize with chemopreventive and chemotherapy agents is essential to assess its chemopreventive/anticancer potential.

Our studies indicated that low-dose digitoxin activated the expression of SRC-mediated signaling pathways, in particular genes induced by ERK1/2 including GRB7, RPS6KA5 and RAB15. High-dose digitoxin activated the expression of stress response and apoptotic genes, specifically, genes activated by the gene EGR-1 [Figure 1c]. These genes included: the ISR transcription factors ATF4 and ATF3, ISR genes PPP1R15A and DNAJB4, apoptotic genes CDKN1A and GDF15 and lipid related genes [Table 4]. Digitoxin decreased expression of the cell cycle related gene CDC16, possibly contributing to arrest in G2/M, and replication gene POLR3B. Real-time RT-PCR validated these findings [Table 5].

As expected, digitoxin altered the expression of several genes involved in calcium homeostasis, including EGR1, IHPK2 and NR4A1 [Table 4]. EGR1, a zinc finger transcription factor, binds to specific GC-rich sequences in the promoter region of target genes that function in growth, and differentiation, thus regulating the expression of genes involved in various signaling cascades.[27] As predicted by its role as a transcription factor, STRING analysis indicated that EGR1 is at the hub of numerous pathways altered by digitoxin treatment. It is noteworthy that EGR1 protein was up-regulated as early as 1 h after treatment with digitoxin at 0.1 *μ*g/ml; since numerous factors can induce the expression of EGR1, it could be a sensitive target for cancer prevention or therapy. Our finding that EGR1 protein, a short-lived phosphoprotein, was induced after treating with digitoxin at 0.1, 0.2 or 1.0 *μ*g/L for one h, and the ATF3 protein was induced after treating with digitoxin at 1.0 *μ*g/ml for 24 h, contradicts the report of Perne *et al*.[15] that digitoxin inhibited general protein synthesis. Further experiments are needed to resolve these differences.

In some systems, EGR1 is thought to be downstream of the ERK pathway.[27] Our results are consistent with the findings that the induction of ATF3 occurs via EGR1 downstream of pSRC and ERK1/2 (MAPK1/2).[26] To test the functional relevance of ERK2, we employed RNAi-mediated gene knockdown; our results suggest that ERK2 mediates the survival response, upstream of EGR-1. Microarray data provide further support for an effect on ERK2 and EGR1: at low-dose digitoxin, increased expression of RPS6KA5, an in vivo substrate for ERKs [Table 3], and, at high-dose digitoxin, increased expression of DUSP8, which specifically inactivates MAP kinase and NAB2. NAB2, in turn, represses EGR1 transcriptional activity [Table 4]. These results are consistent with digitoxin’s inhibition of Na^+^-K^+^-ATPase activity in cardiac myocytes, which activates SRC and downstream ERK signaling cascades that eventually inhibit cell proliferation.[2]

The effects of digitoxin on the expression of genes related to the ISR are not limited to the MDA-MB-453 cell line. Although the MCF7 cell line was less sensitive to the growth inhibitory effect of digitoxon, it exhibited increased expression of ATF4, DDIT3, GDF15, SLC7A11 and CYP1A1 in response to digitoxin treatment. The results of real-time RT-PCR analysis were remarkably similar between the two cell lines [Figure 3, Table 5 and Figure 5]: digitoxin activated early response, cholesterol biosynthetic and ISR genes, depending on the duration of treatment, and progressively induced the expression of apoptotic genes.

Upregulation of lipid biosynthetic genes 6 h after treatment with 0.1, 0.2 or 1.0 *μ*g/ml of digitoxin may be related to the ability of ERK to activate gene transcription mediated by sterols in HepG2 liver cancer cells.[28] This finding is concerning and requires additional research.

The optimal treatment for breast cancer most likely requires a combination of agents or modalities. Digitoxin’s upregulation of ERK resembled the effects of paclitaxel (TAX) in human ovarian SKOV3 cells.[29] At low concentrations (1-100 nM), TAX activated ERK1/2 within 0.5-6 h, whereas the activation was reversed at 24 h or at high concentrations (1-10 *μ*M). Since digitoxin inhibits the Na^+^-K^+^-ATPase, we reasoned that other inhibitors of ERK1 might inhibit the ATPase. We tested the effect of paclitaxel, which is frequently used to treat breast cancer, on the *in vitro* activity of the purified Na^+^-K^+^-ATPase and found it was a potent inhibitor. Consistent with our findings, paclitaxel has been shown to competitively inhibit ATP binding activity of the NTPase/helicase of hepatitis C virus with an IC_50_ of about 22 *μ*M,[30] suggesting that digitoxin and paclitaxel alter different sites on the Na^+^-K^+^-ATPase.

Paclitaxel is known to arrest cells in the M phase of the cell cycle,[31] whereas none of our experiments display an increase in cells in this phase. Paclitaxel and digitoxin thus appear to alter different signaling pathways in the cells. The finding that these two agents have different mechanisms of action may contribute to their ability to exert synergistic effects on cell growth inhibition.

About one-third of invasive breast cancers are ER-negative. It is important to develop novel therapeutic agents which have an acceptable toxicity profile and activity against ER-negative breast cancer. We therefore investigated the growth inhibitory effect of combinations of digitoxin with paclitaxel on Her2 overexpressing, ER low MDA-MB-453 human breast cancer cells and found moderate synergy. The ability of digitoxin to potentiate paclitaxel’s effects at low concentrations on ER-negative human breast cancer cells may permit the use of lower doses of this toxic chemotherapy agent and could delay the development of resistance to paclitaxel in cancer treatment. ER negative, Her2 overexpressing patients have a poorer clinical prognosis, do not benefit from adjuvant hormonal therapy, and are at risk for recurrence and second breast primaries, giving this study more clinical relevance.

## CONCLUSION

It is vital to the future practice of cancer medicine to develop a therapeutic agent that has an acceptable toxicity profile yet is active against estrogen receptor (ER)-negative breast cancer. This is one of the most aggressive cancers and afflicts about a third of the victims of the disease. Furthermore, the optimal treatment for most breast cancers requires a combination of agents. Our work addresses both these requirements.

Our studies indicate the ability of digitoxin to activate transcription of apoptotic factors, repress cell cycle related genes and, at low concentrations, enhance growth inhibition when combined with paclitaxel. Our findings thus suggest that digitoxin, alone and when combined with paclitaxel, is a promising treatment for ER-negative breast cancer. In addition, our findings are the first to alert physicians to the possible dangers to patients who take a combination of digitoxin and paclitaxel. The potential dangers ensuing when paclitaxel and digitoxin are combined are dependent on the dose of digitoxin.
